# Linking clinician interaction and coordination to clinical performance in Patient-Aligned Care Teams

**DOI:** 10.1186/s13012-015-0368-0

**Published:** 2016-01-15

**Authors:** Sylvia J. Hysong, Candice L. Thomas, Christiane Spitzmüller, Amber B. Amspoker, LeChauncy Woodard, Varsha Modi, Aanand D. Naik

**Affiliations:** 1 Center for Innovations in Quality, Effectiveness and Safety, Michael E. DeBakey Veterans Affairs Medical Center, Houston, TX USA; 2 Department of Medicine, Health Services Research Section, Baylor College of Medicine, Houston, TX USA; 3 Department of Psychology, University of Houston, Houston, TX USA

**Keywords:** Coordination, Coordination complexity, Primary care teams, Veterans Affairs

## Abstract

**Background:**

Team coordination within clinical care settings is a critical component of effective patient care. Less is known about the extent, effectiveness, and impact of coordination activities among professionals within VA Patient-Aligned Care Teams (PACTs). This study will address these gaps by describing the specific, fundamental tasks and practices involved in PACT coordination, their impact on performance measures, and the role of coordination task complexity.

**Methods/design:**

First, we will use a web-based survey of coordination practices among 1600 PACTs in the national VHA. Survey findings will characterize PACT coordination practices and assess their association with clinical performance measures. Functional job analysis, using 6–8 subject matter experts who are 3rd and 4th year residents in VA Primary Care rotations, will be utilized to identify the tasks involved in completing clinical performance measures to standard. From this, expert ratings of coordination complexity will be used to determine the level of coordinative complexity required for each of the clinical performance measures drawn from the VA External Peer Review Program (EPRP). For objective 3, data collected from the first two methods will evaluate the effect of clinical complexity on the relationships between measures of PACT coordination and their ratings on the clinical performance measures.

**Discussion:**

Results from this study will support successful implementation of coordinated team-based work in clinical settings by providing knowledge regarding which aspects of care require the most complex levels of coordination and how specific coordination practices impact clinical performance.

**Electronic supplementary material:**

The online version of this article (doi:10.1186/s13012-015-0368-0) contains supplementary material, which is available to authorized users.

## Background

Organizing patient care and enhancing coordination are essential components to improving quality healthcare delivery [1–3]. Many healthcare facilities are transitioning to team-based healthcare where coordination among interdisciplinary team members must occur for successful patient care [4–6]. The VA recently implemented Patient-Aligned Care Teams (PACTs), a team-based adaptation of the patient-centered medical home that has a recommended team configuration consisting of a physician (primary care provider, nurse practitioner, or physician assistant), care manager, clinical associate, and clerical associate [7]. Successful implementation of this new model requires integration of coordination practices into daily clinical and organizational processes and training.

**Fig. 1:**
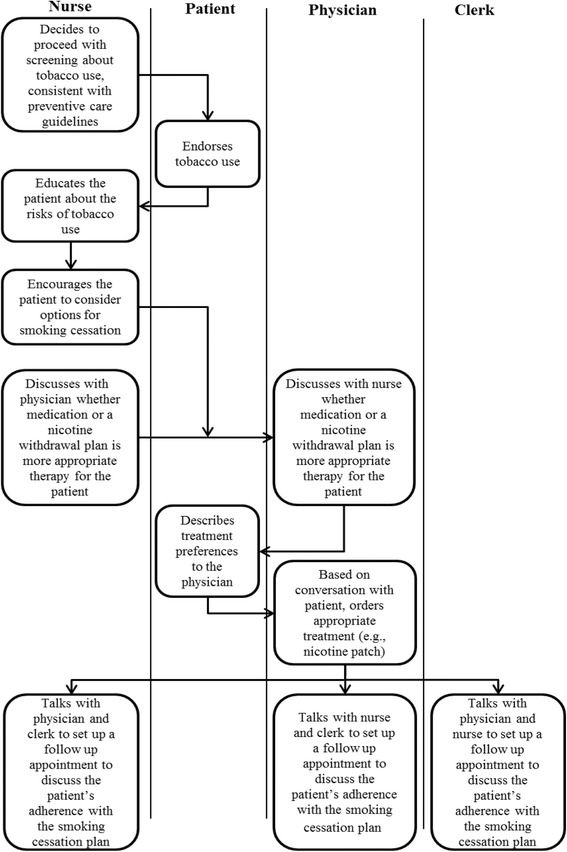
Example process flow of a tobacco cessation screening and therapy coordinative activity performed by outpatient PACTs

One of the defining characteristics of teams is their task interdependence. As a result, coordination is a critical feature of effective teamwork [6, 8] and is a major challenge in the implementation of care teams [9]. Within healthcare, coordination consists of team members working collectively on interdependent tasks and coordination-intensive procedures that require effective communication and task sequencing for these procedures to be completed without errors or delays in care. Figure 1, for example, illustrates the coordinative activities required in tobacco screening and smoking cessation programs

Breakdowns in coordination can give rise to errors that can affect quality, safety, and patient satisfaction [10]. For example, a breakdown in coordination could occur in the activities described in Fig. 1 if it was not clear who was responsible for the discussion of patient preferences. Unfortunately, in some contexts, the implementation of care teams has been associated with clinician overload, role conflict, and decreased job satisfaction due to poor coordination processes [9, 11]. Currently, there is a gap in the literature identifying coordination best practices for healthcare teams. The present study addresses this gap directly, and both describes and measures the enabling factors and influences for coordination as well as the relationship between these enabling factors and effective performance.

### Theoretical foundation

Although many advocate the need for care coordination [2, 12–15], there is little healthcare research examining the evidence-based practices for how successful coordination occurs. To address this gap, we draw on Okhuysen and Bechky’s theoretical model of coordination [8] to facilitate the study objectives of characterizing coordination practices and assessing the association of these coordination practices with clinical performance. This coordination model has been successfully used within healthcare settings [10] and provides an overall framework for detailing the mechanisms and processes and integrating conditions that enable teams to work collectively.

According to this framework, five basic mechanisms underlie effective coordination: plans and rules, objects and representations, roles, routines, and proximity. These five basic mechanisms enable teams to achieve three integrating conditions: (1) accountability (knowing who is responsible for what), (2) predictability (knowing what tasks are involved and when they happen), and (3) common understanding (providing a shared perspective on the whole process and how individuals’ work fits within the whole). It is these three conditions that allow people to collectively accomplish their interdependent tasks [8]. In tobacco screening and therapy, for example (see Fig. 1), effective patient care can only occur if the nurse, physician, and clerk know who is responsible for which task, the sequencing of the tasks, and how each person’s tasks fit within the larger goal of providing smoking cessation guidance.

### Coordination complexity

As different clinical outcomes require a varying number of tasks, and said tasks require different levels of knowledge, skills, and abilities, we expect to find significant variability in the coordinative complexity (i.e., the complexity of interaction needed among personnel to complete the task) required to perform clinical tasks. Within healthcare, coordinative complexity is associated with changes in clinical performance because the higher the task complexity, the more effort or time needed to successfully perform the task [16–18]. Team coordination, therefore, is likely more important for patient care within highly complex situations.

Healthcare quality is likely influenced by the interaction of coordination practices and coordination complexity. For example, the smoking cessation screening process described in Fig. 1 involves 8 steps and 3 clinic personnel whereas a similar screening process for colorectal cancer involves up to 25 steps and 8 personnel [17, 18]. Clinical performance measures, however, often do not take coordinative complexity into account. To best promote successful team implementation, it is necessary to understand the relationship between coordination and performance and evaluate the extent to which the association between these practices and performance varies by the degree of coordinative complexity required for clinical performance outcomes.

### Objectives and hypotheses

We plan to characterize, measure, and evaluate coordination, examine its relationship with clinical performance, and assess the role of coordinative complexity on these relationships. Our study has three objectives and associated hypotheses:

- Objective 1: Characterize coordination practices, and elements of coordination utilized by PACTs (objective 1a) and assess the association between coordination practices utilized by PACTs and clinical performance (objective 1b). We hypothesize that PACTs with a greater number of effective coordination practices (e.g., clear roles, explicit plans, and rules) will have higher levels of clinical performance.
- Objective 2: Determine the level of coordinative complexity required for specific outpatient clinical performance measures. We predict the coordinative complexity required to perform tasks in specific clinical performance measure will vary significantly across measures.
- Objective 3: Evaluate the extent to which the association between coordination and clinical performance varies by the degree of coordinative complexity required to meet each performance measure. We hypothesize that the association between coordination practices and clinical performance will be stronger for clinical performance measures exhibiting higher levels of coordinative complexity.

## Methods/design

Table 1 presents an overview of the methods—a combination of survey methods, focus groups, and analysis of existing data—employed in this study. Methods for each objective are detailed below, followed by a data analysis plan for the entire project.

**Table 1:** Overview of study methods, by objective

Element	Objective 1	Objective 2	Objective 3
Objective	(a) Characterize coordination practices utilized by PACTs and (b) examine their association with clinical performance	Determine the level of coordinative complexity required for each outpatient clinical performance measure	Evaluate the role of measure complexity in how PACT coordination measures relate to EPRP performance measures
Design	Web-based survey of coordination practices	Expert ratings of EPRP measures on coordinative complexity, obtained via functional job analysis (FJA)	Statistical analysis of the impact of coordinative complexity on the association between coordination practices and clinical performance
Participants	Initial validation sample will include teamlet members of 500 PACTs. Full survey deployment will include teamlet members of 1600 PACTs nationwide	6–8 3rd or 4th year residents in VA Primary Care rotations, to serve as subject matter experts (SME)	None. This aim combines datasets from aims 1 and 2
Measures	Outcome: clinical performance, measured by 25 outpatient EPRP measures	Clinical performance: same as objective 1b	Outcome: clinical performance, measured the same as in objectives 1b and 2
	Predictors: coordination practices, measured via web-based survey developed for this project	Coordinative complexity: average Worker Interaction scale ratings for the set of tasks comprising each EPRP measure	Predictor: coordination, measured the same as in objective 1b
	Covariate: PACT integration, calculated with PACT recognition metrics, available via the Patient-Aligned Care Teams Compass Cube		Coordinative complexity ratings: same as objective 2
Procedures	Survey development: coordination survey to be developed and piloted using small groups of PACT teamlet members (*n* = 6–8)	FJA focus groups: SMEs will generate FJA-style lists of task statements comprising the work required to perform each EPRP measure to standard	No new procedures, this aim is strictly analytical
	Survey deployment: web-based deployment via SurveyMonkey, using recruitment strategy recommended by Dillman	Coordinative complexity rating: research team will rate each task on the worker interaction FJA scale	
Data analysis	(a) To evaluate O&B’s measurement model: series of two-level CFAs, followed by EFAs (where fit is unacceptable) and Cronbach’s alphas. To evaluate O&B’s structural model: structural equations modeling (b) hierarchical linear models with PACT level coordination as predictors of clinical performance	No hypothesis tests planned; descriptive statistics for coordinative complexity and number of tasks per EPRP measure	Same as objective 1b, except that we will conduct hierarchical linear models separately for higher versus lower complexity measures and will then compare regression coefficients between models

### Objective 1

#### Design

We will develop, evaluate, and deploy a web-based survey instrument, titled Coordination Practices Survey, to a sample of 1600 PACTs randomly selected from VA Medical Centers (VAMC) nationwide. Survey responses will then be combined with PACT-level clinical performance data from existing VA databases to test study hypotheses.

#### Participants

Participants will consist of members from 1600 PACT teams, selected randomly from approximately 5700 Primary Care PACTs nationwide at all available VAMC (*n*_vamc_ = 150). A PACT teamlet consists of four members: physician (primary care provider, nurse practitioner, or physician assistant), care manager, clinical associate, and clerical associate [7]. To calculate interrater agreement, a minimum of two respondents per team are needed. In previous work with a national sample of primary care providers, we obtained response rates of 50–60 % [19]. To account for these expected response rates as well as exclusion of PACTs due to incomplete data, we will invite 1600 PACTs to participate.

#### Measures and data sources

##### Outcome measure: clinical performance

Clinical performance will be measured using 5 composite outpatient measures (comprised of a total of 25 component measures) from VA’s External Peer Review Program (EPRP), a nationally abstracted database containing performance data for all VA medical facilities on over 90 indicators assessing access, quality of care, cost-effectiveness, and patient satisfaction [20]. Performance measures were selected if they were active, outpatient EPRP measures with data available at the provider level. In cases where one measure is a subset of the other, we only included the parent measure. Table 2 displays the specific outcome measures we propose to study. These measures reflect the collective efforts of the PACTs in providing high-quality care. To accomplish these tasks to standard, all PACT members will be involved to some extent. To obtain a stable estimate of performance, we will collect EPRP data for all facilities for a period from 6 months before deployment of our survey to 6 months after the close of survey.

**Table 2:** Clinical performance measures to be used

Composite measure	Component measures
Behavioral health screening	1. Vets screened annually for major depression dx
	2. Screened positive for depression with timely SRE
	3. PTSD screening using the PC-PTSD at required times
	4. Vets screened for alcohol misuse with score GE 5 with timely brief counseling
	5. Screened pos. at required intervals for PTSD with timely SRE
Diabetes mellitus	6. DM: outpatient—HbA1c annual
	7. DM: HbA1c poor control (OP)
	8. DM: BP LT 140/90 (OP)
	9. DM: retinal exam, timely by disease (OP)
	10. DM: renal testing (OP)
Ischemic heart	11. IHD LDL-C LT 100 or mod dose statin (OP)
	12. HTN: Dx HTN and DM with BP less than 140/90 (OP)
	13. HTN: Dx HTN and no DM with BP less than 150/90 (OP)
	14. HTN: outpatient BP < 140/90 ages 18–59
Prevention	15. Obese patients screened and offered weight management
	16. Pneumococcal immunizations (OP)
	17. Influenza immunizations age GE 65 (OP)
	18. Influenza immunization 18–64 (OP)
	19. Breast cancer screening women 50–74 years (OP)
	20. Cervical cancer screening women ages 21–29 years
	21. Cervical cancer screening women ages 30–64
	22. Colorectal cancer screening ages 50–75
Tobacco	23. Patients using tobacco offered meds (OP)
	24. Patients using tobacco provided with counsel (OP)
	25. Patients using tobacco offered referral (OP)

##### Independent variables: coordination practices

Coordination will be measured via the Coordination Practices Survey, an online survey created specifically for this study (see the “Procedure” section).

##### Covariate: PACT integration

Although PACTs were implemented nationwide, facilities had some discretion in their implementation; therefore, the degree to which facilities have fully implemented PACTs will likely vary. To account for this potential confounding effect of PACT implementation, we will use a score that describes the level of integration to risk adjust for PACT integration variability. We define this construct as the degree to which facilities and their PACT teams have put in place features—such as same-day access, telephone utilization, 2-day post-discharge contact, and patient continuity with provider—conducive to the team-based model of care. To measure PACT integration, we will calculate a score for each PACT using the PACT Recognition methodology employed by VA’s Office of Primary Care Operations [21], which uses PACT recognition metrics maintained by the Office of Primary Care Operations (OPCO), and available on the PACT Compass Cube hosted by the VHA Support Service Center (VSSC).

The PACT Compass Cube provides a series of metrics that reflects the dimensions and principles of the PACT to indicate whether a facility is on the right path in integrating PACT teams. The metrics in the compass—e.g., non-traditional care modalities, access metrics, continuity metrics, and coordination of care metrics—are based on patients assigned in the Primary Care Management Module (PCMM) to a team and primary care provider. The computations for the PACT integration and the individual recognition metrics we will employ have been used nationally by the VA and are recognized by the VA leadership.

##### Participant characteristics and contact information

After completing all necessary VA Data User Agreements, we will request and merge two datasets, VA’s PCMM and the Personnel Accounting Integrated Database (PAID), to help us identify potential survey participants. The PCMM report displays, by facility, primary care staff members and team positions assigned to all active PACTs. We will use the Full Time Equivalent (FTE) field in the database to determine PACT eligibility: team providers are required to have a FTE of ≥0.80 to be included in the participant sample pool. PAID, the VA’s national employee database, will provide information on employment site, job title, length of employment, PACT assignment date, and contact information including PACT members’ email addresses.

#### Procedure

##### Coordination practices survey development and validation

To develop scales to measure coordination, we will follow DeVellis’ recommendations for scale development [22], considered by survey developers as highly rigorous. The following steps will be used:

1. *Construct clear, operationalizable definitions of our constructs.* To prepare for this, we have crafted preliminary, context-free definitions based on our conceptual model of coordination. These operationalizations will be tailored to the PACT setting with the aid of PACT providers and research collaborators experienced in coordination and team research.
2. *Develop an item pool that maps onto each construct.* Based on the operational definitions defined in step 1, we will search the literature for appropriate scales and items and construct new items where gaps are present. We will tailor our preliminary items to the PACT setting and create new items to achieve a goal of 4–7 items per construct. Response choice format will be Likert scaled (i.e., ranging from strongly disagree to strongly agree, using a five-point scale).
3. *Test survey for usability and clarity.* The items will then be tested with an independent set of 6–8 local VA PACT member participants who will provide suggestions regarding item modifications. Starting with the first participant, each will complete a talk-aloud procedure while completing the survey, describing their reactions and questions to each item. The survey will then be refined based on the participant’s comments, and a new version will be presented to the next participant. In finalizing the survey instrument, we will ascertain that survey completion duration will not exceed 15 min and that all relevant constructs are measured with at least four items per construct.
4. *Test the factor structure and reliability of the O&B model.* We will invite 500 PACTs to complete the survey as a validation sample and conduct psychometric analyses (see the Data analysis plan (all aims) section) to evaluate the reliability [23] and factor structure of the instrument. Recruitment procedures for the validation sample will mirror those of the main study sample.

##### Contacting eligible participants

Adapting Dillman’s recommendations [24] to recruit participants and deploy the survey, we will (a) contact, via email, the Facility Medical Directors and Associates Chief of Staff of Research at each facility to notify them of the survey effort; (b) contact the 1600 PACT teamlets via email 2 weeks prior to survey deployment and inform them of the proposed research study; and (c) send formal survey invitations to the 1600 PACTs. Actual survey invitations will be deployed via email and will contain an individualized hyperlink to the survey.

##### Deploy online survey

The online survey will be created and deployed using REDCap, a VA approved online survey platform managed by the Vanderbilt University Office of Research and hosted locally by the VA Information Resource Center (VIReC) [25]. To ensure data security, the VA’s implementation of this platform functions behind the VA firewall and restricts access to only VA employees. We will send follow-up emails to non-responders 2 and 3 weeks after the initial survey invitations are sent. After 3 weeks, individuals who have not responded will be contacted via telephone. The survey will remain open for a period of 4 weeks, up to 8 if necessary.

### Objective 2

#### Design

This aim will use expert ratings of existing quality measures obtained via functional job analysis (FJA).

#### Methodology overview—understanding FJA

Before rating the coordinative complexity of each outcome, a structured list is needed of the tasks required to perform each measure to standard. We will use functional job analysis (FJA), a task-oriented methodology commonly utilized for systematically describing the complexity of work, to accomplish this. FJA methodology and its use in healthcare have been extensively documented elsewhere [26–29] and is therefore only briefly described here. FJA uses task statements as the basic building blocks of human resource management and organizational strategic planning. Task statements describe the work content, worker characteristics, and the work organization [26]. Once developed, these tasks can then be rated on the complexity of worker interaction, providing a measure of coordination complexity.

In an FJA, tasks required to complete a performance measure to standard are identified through subject matter expert (SME) input. It is from the information in these task statements that ratings of coordinative complexity will be assigned to each EPRP measure. Once the requisite tasks are generated and edited to conform to the standard FJA linguistic and format requirements, they are rated according to their complexity with respect to the functional skill dimension worker interaction, which rates each task on the complexity of interaction needed among personnel to complete the task.

#### Participants

SMEs for the FJA will consist of eight 3rd and 4th year residents in VA Primary Care rotations that have been identified by collaborating physicians as having experience with the clinical work involved in satisfying the EPRP measures used in this study.

#### Measures

##### Clinical performance

The measures of clinical performance to be rated will be the same as those used in objective 1.

##### Coordinative complexity

Coordinative complexity is conceptualized here as the complexity of interactions among personnel in a clinical performance measure’s component tasks. Coordinative complexity for each EPRP measure will be calculated as the average of the Worker Interaction scale ratings for the set of tasks comprising each EPRP measure (See Additional file 1 for the scale). We will be assessing a total of 25 measures of clinical coordinative complexity. Ratings for 17 of the 25 measures proposed were developed in earlier pilot work; thus, ratings for only 8 of the EPRP measures are needed. An important feature of coordinative complexity is that it is considered a static characteristic of each clinical performance measure; coordinative complexity ratings should be invariant across individuals or sites because it is the work, not the worker or the work environment, that is complex or simple.

#### Procedure

##### Identifying the work requirements for each performance measure

To identify the set of tasks required to accomplish each EPRP measure, we will use FJA. The SMEs will receive a document containing each EPRP measure and its definition according to the EPRP Technical Manual. For each measure, the SMEs will independently list all of the tasks required to satisfy the criteria for the measure. SMEs’ responses will then be compiled into a single list of tasks each measure. The resulting tasks will be cross-checked against a validated database of primary care tasks created using FJA as well as the task bank created from our pilot work [27].

Tasks in the task bank follow a structured format and have been rated on coordinative complexity. For tasks listed by the SMEs with matching tasks in either database, we will use the validated task and its ratings. Tasks without a database match will be generated in a series of focus groups with the SMEs and will conform to FJA task structure and format. For each unmatched task, SMEs will describe the specific behaviors and actions, knowledge, skills, abilities, and tools required to achieve the result listed in the task. Tasks generated from the focus group will then be edited as needed to ensure they conform to FJA structure. The SMEs will review the final set of tasks for each measure to confirm whether the tasks associated with each measure comprise at least 85 % of the work required to accomplish the measure to criterion [26], to ensure the task bank is sufficiently complete to cover the set of tasks performed in the EPRP measures [29].

##### Rating the performance measures on coordinative complexity

Two independent raters from the research team will rate each newly generated task on coordinative complexity using the standardized scale prescribed by FJA. Discrepancies in ratings will be resolved by consensus. We will average the coordinative complexity ratings across all tasks within an EPRP measure to arrive at a measure-level rating of coordinative complexity.

### Objective 3

#### Participants, measures, and procedure

To examine the effect of coordinative complexity on the association between coordination and clinical performance, the same datasets, measures, and participants from objective 1 will be used in conjunction with the coordinative complexity ratings generated in objective 2.

### Data analysis plan (all aims)

#### Sample representativeness

We will perform comparisons to evaluate the representativeness of our sample. First, we will compare the sample’s distribution along several demographic characteristics (age, gender, race, years in VA, and clinical specialty for providers) to the distribution of the population of VA Primary Care Clinics using the *χ* ^2^ Goodness-of-Fit test and *t* tests, where appropriate. Next, we will compare the sample’s distribution to the distribution of all PACT team members invited along the same demographic characteristics. These comparisons in concert will provide a more nuanced picture of representativeness than a simple response rate. In addition, to check for the representativeness of the PACTs themselves, we will use the *χ* ^2^ Goodness-of-Fit test to compare the sample’s distribution of PACT configurations to that of the overall VA PACT population. Finally, we will compare the distribution of facility characteristics of all VAMCs to those of VAMCs whose teams responded to our survey.

#### Survey psychometric evaluation

We will use the 500 PACTs from the validation sample to assess preliminary psychometric evaluations of the items designed to measure Okhuysen and Bechky’s coordination constructs. We will then use the data from the full survey deployment to 1600 PACTs as a cross validation for the derived model based on the smaller validation sample and conduct a two-level multilevel confirmatory factor analysis (CFA) with maximum likelihood estimation to determine the fit of the model to the larger sample. The intraclass correlation coefficient (ICC) will be calculated to examine the dependency of PACTs within VAs. Assuming the ICC is significant, a two-level multilevel CFA will be conducted with maximum likelihood estimation to determine the fit of the items to the theorized coordination constructs. We are primarily interested in the fit of the level-one model (i.e., the PACTs).

If the model fit statistics are not acceptable (e.g., CFI or TLI < 0.95, RMSEA or SRMR > 0.05), we will conduct a multilevel exploratory factor analysis using oblique rotation overall and within each content area to examine dimensionality, using parallel analysis to determine the appropriate number of factors [30, 31]. The simple structure of each item will then be examined and will aid in determining which particular items should be retained in a given factor. Each construct is expected to be unidimensional. However, if multiple dimensions emerge, they will be reviewed for interpretability and sub-dimensions may be created. We will compute Cronbach’s alpha for each factor/dimension to evaluate the internal consistency reliability. After evaluating the measurement model for Okhuysen and Bechky’s coordination constructs, we will then estimate direct and indirect effects to test the theoretically derived structural model of team coordination. In brief, coordinating mechanisms (i.e., plans and rules) are thought to predict integrating conditions (i.e., accountability, predictability, common understanding) through a variety of coordination processes.

#### PACT-level aggregation

The first step in identifying PACT-level coordination practices is aggregating individual survey responses to the PACT level. Commonly, item responses from members of each team are averaged to obtain a group level estimate of each individual item and then average those responses to form the scale. In the case of an inherently collective construct such as coordination, however, differences in individual responses do not constitute measurement error but rather real variability and a valuable component of the construct. To capture this phenomenon in the scores, we will calculate *r*_wg_, interrater agreement, for each coordination construct for each PACT, and weight each PACT’s coordination construct scores by their respective *r*_wg_ scores. *r*_wg_ indices range from 0 to 1 (1 indicating perfectly homogeneous within-PACT scores on an item) [32].

EPRP measurements from 6 months prior to 6 months after deployment of the survey will be averaged together to create a single representative measure of performance. This will provide a more stable estimate of clinical performance than a single quarter’s data.

#### Descriptive statistics and preliminary diagnostics

Preliminary summary statistics will be calculated for the coordination practices, coordinative complexity, and PACT integration variables to investigate characteristics such as distributional properties, presence of ceiling or floor effects, and missing data. We will examine the data for missing values and use multiple imputation procedures to replace, if necessary, the missing data with values that reflect the uncertainty about which values to impute.

#### Hypothesis tests

We will use an analysis of variance test or Kruskal-Wallis test (if residuals are not normally distributed) to evaluate differences in coordinative complexity between existing outpatient EPRP measures (objective 2). Objectives 1b and 3 will both be evaluated with hierarchical linear modeling [33]. Two-level hierarchical linear models will be constructed, with PACTs nested within facilities. The PACT’s average EPRP score will be the dependent variable, and coordination constructs from the survey will be level 1 (between-PACT) independent variables. We will only include the most downstream endogenous coordination variables as predictors in our models. Although it depends on the psychometric evaluation of Okhuysen and Bechky’s model described above, integrating conditions (i.e., accountability, predictability, common understanding) are theoretically the most important coordination constructs that all other constructs lead to and, therefore, we anticipate they will be the independent variables in our models. PACT integration will be included as a level 1 covariate. Objective 1b will be supported if coordination practices significantly predict clinical performance.

In order to test objective 3, we will first group performance measures into those with varying levels of (e.g., higher versus lower) coordinative complexity. We will then conduct a two-level hierarchical linear models (identical to the one described above) for each level of coordinative complexity and will statistically compare regression coefficients. As before, models will include PACT integration as a covariate. Hypothesis 3 will be supported if coordination complexity is more strongly related to performance among measures that have higher coordinative complexity.

### Study status

The instrument is currently developed and ready for deployment, clinical performance metrics selected, and potential participants identified. We are currently in the process of piloting the survey for psychometric soundness before beginning principal data collection.

## Discussion

### Contributions to practice

Addressing the current gaps in our understanding of coordination within clinical care settings, this study will provide an in-depth look at the specific fundamental practices involved in coordination, their impact on clinical outcomes, and the role of coordinative complexity in these relationships. Understanding which aspects of care require the most complex levels of coordination and how specific coordination practices are associated with clinical performance will allow for maximization of the efficacy of the implementation of coordinated team-based work. The results of this study will aid the implementation of the care teams by elucidating which coordination practices are most likely to result in improved care.

### Contributions to science

Assessing coordination within a large sample of teams allows us to simultaneously test all aspects of coordination; this will constitute a material contribution to the literature as coordination, and team-based research often suffers from small sample sizes. To our knowledge, this will be the first empirical test of this comprehensive coordination framework as a whole.

### Limitations

Although we utilize longitudinal multi-source data to assess our objectives, there are several limitations of the proposed design. First, the population of outpatient primary care providers at the VA were selected due to the recent implementation of PACTS; however, this restriction limits the generalizability of the results and future research will be needed to assess the applicability of the results to other healthcare facilities and types of care. Second, although we implement a validation sample to help in the creation and psychometric assessment of scales, the coordination literature lacks clear measurement strategies and developed scales. Therefore, although we preliminarily assess the validity of the scales utilized, the dimensionality and validity inferences are limited to the sample of VA care providers and will need to be replicated in other samples to comprehensively understand the psychometric utility of these scales.

### Ethics, consent, and permissions

This study was reviewed and approved for ethics compliance by Baylor College of Medicine’s Institutional Review Board (BCM IRB; H-30952). Consent to participate and consent to publish are not applicable for this study—no individual information or participants will be utilized. Participants in the survey portion of the study will be asked to provide informed consent, using a BCM IRB-approved consent form, to participate, prior to access to the survey link. No individual data will be reported or published.

## Additional file

Additional file 1:
**Description and definitions for Worker Interaction Scale used to measure coordinative complexity.** (PDF 77 kb)
