# Supplementary material for: Linking clinician interaction and coordination to clinical performance in Patient-Aligned Care Teams
Source: Implement Sci. 2016 Jan 15;11:7. doi: 10.1186/s13012-015-0368-0 (PMC4714534; doi:10.1186/s13012-015-0368-0)
Supplement: Additional file 1: — Description and definitions for Worker Interaction Scale used to measure coordinative complexity. (PDF 77 kb) [file 13012_2015_368_MOESM1_ESM.pdf]

Additional File 1 – Worker Interaction Scale

Description of worker interaction: When working with others, through direct or indirect contact, workers assist them, coordinate their efforts with them and adapt their style and behavior to accommodate atypical or unusual circumstances and conditions. This effort results in achievement of employer goals to given standards.

Level	Definition	Detailed description/Example
1	Solitary Work	Worker is required to operate independently of contact with others
2	Unilateral Assistance	Worker is required to receive from, or give to, others the communication or resources required to complete the unit of work
3	Bilateral Assistance	Worker is required to receive and return the communication and/or resources from others required to complete the unit of work
4	Behavioral Coordination	Worker is required to expend discretionary time and effort within standard operating procedures to coordinate behavior with that of others
5	Behavioral Adaptation	Worker is required to modify and improvise behavior within existing management or systems parameters in adapting to the needs and capabilities of others
6	Systems Adaptation	Worker is required to develop or change the routines which define and delimit expected social-organizational interactions with others

References

1. Fine SA, Cronshaw SF: Functional Job Analysis: a Foundation for Human Resources Management. Mahwah, New Jersey: 1995.
2. Cronshaw SF & Alfieri AJ: The impact of sociotechnical task demands on use of worker discretion and functional skill. Human relations, 2003, *56*(9): 1107-1130.
